# Poverty dynamics and the determining factors among East African smallholder farmers

**DOI:** 10.1016/j.agsy.2023.103611

**Published:** 2023-03

**Authors:** James Hammond, Tim Pagella, Mark E. Caulfield, Simon Fraval, Nils Teufel, Jannike Wichern, Esther Kihoro, Mario Herrero, Todd S. Rosenstock, Mark T. van Wijk

**Affiliations:** a International Livestock Research Institute, Nairobi, Kenya; b Bangor University, Bangor, Wales, United Kingdom; c The University of Edinburgh, Edinburgh, Scotland, United Kingdom; d Rhine-Waal University of Applied Sciences, Kleve, Germany; e Department of Global Development, College of Agriculture and Life Sciences and Cornell Atkinson Center for Sustainability, Cornell University, Ithaca, NY 14850, USA; f Alliance of Bioversity and CIAT, Nairobi, Kenya

**Keywords:** Agricultural intensification, Off-farm, Food security, Poverty dynamics, Smallholders, East Africa

## Abstract

**CONTEXT:**

Rapid economic development in East Africa is matched by extremely dynamic smallholder livelihoods.

**Objective:**

To quantify the changes in poverty of smallholder farmers, to evaluate the potential of farm and off-farm activities to alleviate poverty, and to evaluate the potential barriers to poverty alleviation.

**METHODS:**

The analyses were based on a panel survey of 600 households undertaken in 2012 and re-visited approximately four years later in four sites in East Africa. The sites represented contrasting smallholder farming systems, linked to urban centres undergoing rapid economic and social change (Nairobi, Kampala, Kisumu, and Dar-es-Salaam). The surveys assessed farm management, farm productivity, livelihoods, and various measures of household welfare.

**RESULTS AND CONCLUSIONS:**

Almost two thirds of households rose above or fell below meaningful poverty thresholds – more than previously measured in this context – but overall poverty rates remained constant. Enhanced farm value production and off-farm income proved to be important mechanisms to rise out of poverty for households that were already resource-endowed. However, households in the poorest stratum in both panels appeared to be stuck in a poverty trap. They owned significantly fewer productive assets in the first panel compared to other groups (land and livestock), and these baseline assets were found to be positively correlated with farm income in the second panel survey. Equally these households were also found to be among the least educated, while education was found to be an important enabling factor for the generation of high value off-farm income.

**SIGNIFICANCE:**

Rural development that aims to stimulate increases in farm produce value as a means to alleviate poverty are only viable for already resource-endowed households, as they have the capacity to enhance farm value production. Conversely, the alleviation of extreme poverty should focus on different means, perhaps cash transfers, or the development of more sophisticated social safety nets. Furthermore, while off-farm income presents another important mechanism for poverty alleviation in rural areas, these opportunities are restricted to those households that have had access to education. As more households turn to off-farm activities to supplement or replace their livelihoods, farming approaches will also change affecting the management of natural resources. These dynamics ought to be better understood to better manage land-use transitions.

## Highlights

• A panel survey of 600 households was undertaken around four years apart in four sites in East Africa
• Enhanced farm value production as a pathway out of poverty was only open to resource-endowed households
• The participation in high-value off-farm income as a pathway out of poverty was only open to educated households
• Households participating in low-value off-farm activities were trapped in a vicious cycle of diminishing resources
• Intensification efforts may support medium-poverty households, alleviation of extreme poverty should be by different means

## Graphical abstract

**Figure:**
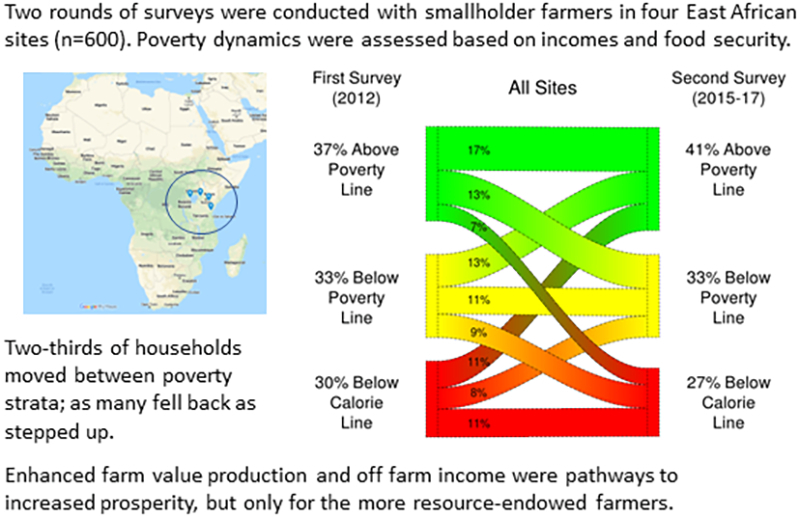

## 1 Introduction

Despite decades of work to understand and eradicate poverty in rural sub-Saharan Africa, there is still a paucity of data on the links between smallholder farming practices, their livelihoods, and poverty dynamics (Carletto et al., 2015). Enhanced farm production is widely viewed as one major route to increasing food production and thus improving food security (Foley et al., 2011), alleviating rural poverty at the household level⁠, and as an engine of the economy at the national level (Ejeta, 2010; Larson et al., 2016). An alternative – or perhaps complementary – pathway out of poverty and toward greater food security is presented by off-farm income activities.

The degree to which enhanced farm value production, either through agricultural intensification or the substitution of lower value products with higher value products, can alleviate smallholder poverty has been called into question through farm system modelling. For example, in two studies on the potential of agricultural intensification to lift rural households out of poverty in sub-Saharan Africa, it was found that a ten- to one-hundred-fold increase in income per hectare would be required (Carletto et al., 2015; Harris and Orr, 2014). In another farm optimisation modelling study from Mozambique, it was found that agricultural intensification was only feasible for wealthier farming households (Leonardo et al., 2018), while a simulation-modelling study from western Kenya found that there was a farm-size threshold of around 0.4 ha before yield intensification could lead to important household income gains (Waithaka et al., 2006).

Household and field level studies have also found a number of reasons why farmers often cannot benefit from intensification practices, explaining the lack of upscaling of these commonly promoted techniques (Baudron et al., 2012; Raut et al., 2011). For example, in a study from Ghana and Malawi using semi-structured interviews, it was found that poor access to land and gender-biased land tenure systems were a main factor in preventing farming households from employing sustainable agricultural intensification techniques (Fischer et al., 2020). The lack of responsiveness of soils as a result of land degradation has also been ear-marked as an important deterrent to the increased use of these techniques (Burke et al., 2017; Liverpool-Tasie et al., 2017; Vanlauwe et al., 2011). These factors can often lead to “poverty traps”, where a lack of investment in soil fertility leads to declining yields, and thereby fewer resources with which to invest in techniques to enhance agricultural intensification (Tittonell and Giller, 2013).

The role of rural mobility and the generation of income through off-farm activities has also been shown to have important relationships with rural poverty alleviation (Caulfield et al., 2021; Duong et al., 2021; Rakotoarisoa and Kaitibie, 2019). Indeed for many households in sub-Saharan Africa, the role of off-farm income has been identified to be in a similar magnitude of importance as agricultural incomes (Frelat et al., 2016; Reardon, 1997; Ritzema et al., 2017).

These relationships however are complex, mediated by different contextual factors (Caulfield et al., 2021). In some cases it has been found that high-value off-farm income generation has been associated with the greater use of agricultural intensification techniques, such as high-yielding seed technologies (Mendola, 2008) or the use of agro-chemicals (Gray, 2009). In other instances, off-farm income has been found to be associated with farming de-intensification as households generate more income through their off-farm activities (Qin, 2010). While these instances highlight how high-value off-farm income may secure rural household livelihoods through rural mobility enabling them to remain in their rural communities (Mata-Codesal, 2018; Ye, 2018), other studies reveal that more vulnerable households are obliged to turn to low-value off-farm income activities to supplement their farm income in order to survive (Alary et al., 2022; Barrett et al., 2001). In these latter cases, the labour losses experienced as a result of engagement in low-value off-farm income activities further constrains the options available to these rural households to lift themselves out of poverty (Sarker et al., 2020).

The objective of our study was therefore to quantify the changes in the material prosperity/poverty of smallholder farmers through time and evaluate the role of farm and off-farm activities in this process. To do so we present the results of a household panel survey exercise in which 600 households were visited in 2012 and re-visited approximately four years later (from late 2015 to early 2017, depending on the location), in four sites in East Africa. All are linked to urban centres undergoing rapid economic and social change (Nairobi, Kisumu, Kampala and Dar-es-Salaam), through transport infrastructure, markets, and opportunities for off-farm incomes. Comprehensive data on farm management, farm productivity, livelihoods, and various measures of household welfare were gathered. This unique dataset allows us to study smallholder poverty dynamics in relation to farm value production and off-farm income, in settings representative for many developing centres across sub-Saharan Africa. We build on recent analytical techniques applied so far only to single time-point studies (Frelat et al., 2016). We hypothesised that enhanced farm value production as a means to increase household income from one time point to the next would be observable for households that were more resource-endowed (with financial and land assets), but would be less likely in households with lower resource endowment. Moreover, we hypothesised that off-farm income activities also presented a pathway for households to enhance their overall income, but this pathway was also constrained by household characteristics such as education attainment.

## 2 Materials and methods

### 2.1 Site descriptions

Four sites were selected in East Africa: Lushoto in Tanzania, Rakai in Uganda, and Wote and Nyando in Kenya. All four were benchmark sites of the Climate Change, Agriculture and Food Security (CCAFS) program of the CGIAR and the Earth System Science Partnership. It was notable that during the second phase of data collection, East Africa experienced severe drought conditions in 2016 associated with climate change and El Niño (Uhe et al., 2018); otherwise the climate was fairly stable and market conditions good.

Lushoto, Tanzania is characterized by mixed farming, with vegetable production for sales to Dar-es-Salaam playing an important role in recent years (Fraval et al., 2018). The site ranges in elevation from 780 to 2010 m above sea level. Rainfall is bimodal, ranging from 690 to 1230 mm per year. Population density was 120 people per km^2^ in 2012. Of the 200 households surveyed in 2012, 147 randomly chosen households (73% of the households from 2012) were resurveyed in 2015.

Rakai, southern Uganda, is a site with a steep rainfall gradient, with high rainfalls (>1400 mm) along Lake Victoria, rapidly declining to low into Western Rakai and Isingiro (<1000 mm). Population density was 154 people per km^2^ in 2014. The production targeted is characterized as a mixed coffee–banana system with annuals and few local livestock included. Of the 200 households surveyed in 2012, 135 randomly chosen households (68% of the households from 2012) were resurveyed in early 2017.

Wote, Eastern Kenya, is the driest site of the four sites included in this study with an average rainfall of 520 mm per year. Rainfall is bimodal, with the main cropping season in the later rains. Population density was 110 people per km^2^ in 2009. Two mixed farming systems are common in the area, crops mixed with local sheep and crops mixed with dairy cows. Key crops in the region are sorghum and millet, cow pea and pigeon pea. Of the 200 households surveyed in 2012, 160 randomly chosen households (80% of the households from 2012) were resurveyed in 2016.

Nyando, Western Kenya, is characterized by a mixed crop-livestock system. Annual rainfall is highly variable, ranging between 400 and 750 mm per year. Population density was 341 people per km^2^ in 2009. Maize, sorghum and beans are the three most important crops in this area. Of the 200 households surveyed in 2012, 160 randomly chosen households (80% of the households from 2012) were resurveyed in 2016.

### 2.2 Sampling and survey tools used

The four sites were defined as rectangular blocks of land measuring approximately 10 km by 10 km. Once the blocks were chosen and mapped, all villages within the block were enumerated and seven villages were randomly chosen within the block, and in turn 20 households within each village were randomly chosen. For the second set of interviews, a subset of the households was randomly identified from household lists, and non-available households were substituted with other households from the list of those originally interviewed in 2012.

In the first panel round the Impact-Lite tool was used. The tool was designed to capture in detail agricultural practices and has been described in detail elsewhere (Rufino et al., 2013)⁠. For the latter round of household surveys, a different survey tool was used: the Rural Household Multi-Indicator Survey (RHoMIS) (Hammond et al., 2017; van Wijk et al., 2020)⁠. The use of different survey tools for the two panels was a result of project decisions to enhance efficiencies in the collection of survey data, with the latter survey (RHoMIS) taking less time to implement than the former (ImpactLite). Nevertheless, both surveys captured the full inventory of crops and livestock raised over the past 12 months, including products yielded, consumed and sold. Off-farm incomes were also covered. All data relied on interviewees to recall and report on their household's characteristics, land-use, crop and livestock production and sales. With the raw information collected by both survey tools we could quantify land areas by crop (proportion of overall land-use), crop production and yield (based on overall annual crop production and land-used), livestock production (based on livestock owned by species), consumption (based on proportion consumed and proportion sold) and sales of farm produce (based on reported sales income from main crops and livestock – see Table S1 for an overview of prices by site and panel), on and off-farm income (based on previous calculations and figures), and compare changes in these variables over time. To calculate quantities per capita, “male adult equivalent” household size was used to allow the direct comparison between households of different sizes and compositions (Weisell and Dop, 2012).

The RHoMIS questionnaire was also designed to capture various indicators of household welfare and food security: the Household Food Insecurity of Access Scale (HFIAS) (Coates et al., 2007)⁠ which measures the frequency and severity of hunger, the Household Dietary Diversity Score (HDDS) which provides an indication of household nutrition status (Swindale and Bilinsky, 2006)⁠ but using more recent and more nutritionally relevant food group categories (FAO and FHI 360, 2016)⁠, and the Probability of Poverty Index (PPI) (Desiere et al., 2015)⁠, which is an asset-based scoring system to estimate the likelihood that a household is in poverty.

### 2.3 Data processing and analysis

To compare between the two panel survey rounds, we calculated three poverty classes using two thresholds: the lower threshold representing the ability of a household to secure adequate calories per person (3000kCal), and the upper threshold set at the total value of produce, sales, and incomes of $1.90 per person per day, using World Bank 2015 purchasing parity power conversion rates. The approach is derived from the Food Availability indicator, which was found to be useful and reliable in many locations in Sub-Saharan Africa (Frelat et al., 2016; Ritzema et al., 2017). In this indicator all consumed farm produce (expressed in calorie equivalent) are combined with the money earned by sales of produce and off-farm income. Cash values are converted into calorie values at local market prices for staple foodstuffs (in this case maize). This resulted in three levels of poverty in each panel round: the lowest level of households unlikely to be able to supply their basic calorie needs, the middle level of households able to supply their basic calorie needs but still living below the international poverty line, and the highest level of households living above the international poverty line. The poverty groups were then validated against welfare indicators gathered in the latter panel and based on data not used to establish the poverty grouping. These were the household dietary diversity score in the flush and lean seasons, the household food insecurity of access scale, the number of months with food shortages, and the probability of poverty index (see section 2.2). The values of each of these three indicators were compared between the three poverty strata, with the hypothesis that the higher strata should score better on the indicators borne out (*p* *<* *0.05*; see Supplementary Information Fig. S1 and Table S2). This validation was important in order to prove that the categories and trajectories identified using in total value of activities were fundamentally related to overall household wellbeing.

The poverty groups in both panels were compared to identify distinguishing characteristics of more or less prosperous households at each time point, and to investigate whether these characteristics changed over the four-year period. Changes to overall poverty rates were analysed using McNemar's Chi-squared test, which permits analysis of paired data. The households were then sorted into trajectory groups, according to the (change in the) group to which the household belonged in each of the two panel survey rounds. Households' movement between poverty groups was analysed using the Wilcoxon Signed Rank test. We analysed which variables were associated with different trajectories, and the direction and magnitude of change of those variables: we analysed productive assets (land and livestock holdings, aggregated as TLU (Njuki et al., 2011)⁠) and measures of farm performance (production, consumption, sales, and off-farm income) and the number of household members. To assess value production intensification, three efficiency indicators were used: value of crop production per hectare of land, value of livestock production per unit of livestock, and off-farm income per male adult equivalent. The denominators used for these indicators (per area of land, per livestock unit, and per male adult equivalent respectively) were selected based on the output unit with which the productive asset was most closely associated and the ones most commonly used in the scientific literature. It could have been possible to convert the units to a common denominator (eg, per male adult equivalent) to enhance comparability within the current study, however, it was decided that comparability with production figures from other studies was of greater utility. Significant differences between subsets of households were analysed using ANOVA, Tukey's Honest Significant Difference, and Fisher's Least Significant Difference posthoc tests. Outliers were excluded from boxplots, for ease of visualisation, but were included in testing of significant differences. Linear regression analyses were used to test the relationship between productive assets and farm income (USD$ year-1). ANOVA and a Fisher's Least Significant Difference posthoc tests were used to assess the relationship between education level attainment on-farm and off-farm income.

## 3 Results

### 3.1 Comparing between the panels

#### 3.1.1 The prosperity strata

**Fig. 1:**
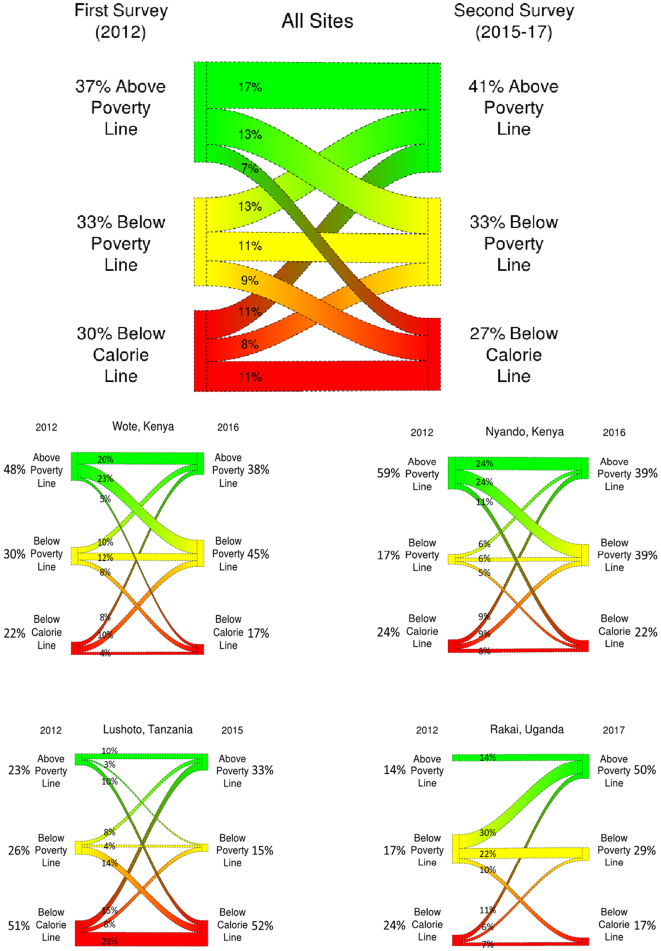
The proportions of households above and below the $1.90 poverty line, and above and below a calorie line set at 3000 kCal per male adult equivalent person, for both panel survey rounds. The proportions of the study sample moving between groups are also shown. Fig. 1A shows the study population, Fig. 1B–E show results per site Wote, Kenya (160 households), Nyando, Kenya (160 households), Lushoto, Tanzania (147 households), and Rakai, Uganda (135 households) respectively.

Fig. 1A (and Fig. 1B to E per site) present the proportion of households in each prosperity strata in each panel, as well as the change in proportion of households from one stratum to another. Fig. 1 shows that a majority of households were either rising or falling between strata, although the total number of households in each of the three poverty strata did not show a significant difference (according to the McNemar Chi-Squared test between the first and second panel survey, when considering the whole study population). In the earlier panel, 30% of households were classed in the lowest stratum, 33% in medium and 37% in high. In the second panel, 27% were classed in the low stratum, 33% medium and 41% high.

When examined individually, the sites each showed significant changes in poverty rates. In Nyando and Wote (Kenya) more households dropped below the poverty line than rose above it (*p* < 0.001 and *p* < 0.05 respectively). Lushoto (Tanzania) showed the most static system, and the most households below the calorie line, although there were more households rising than falling (*p* = 0.06). Rakai (Uganda) showed the most positive situation with the most household rising above the poverty line (p < 0.001).

To establish confidence in the identified strata, five independent indicators of household welfare were calculated per poverty strata group, for the latter panel (unfortunately data for these indicators were unavailable for the first panel). Households in more prosperous classes showed significantly lower likelihood of being in poverty, according to the probability of poverty index score, lower number of hungry months, lower reported household food insecurity of access score, and a higher household dietary diversity score in both the post-harvest good season and the lean season (see Fig. S1 and Table S2; Tukey HSD *p* *<* *0.05*) thereby confirming the expected overall welfare differences between groups.

#### 3.1.2 Land, labour and livestock

In both panels, households were typically composed of 5 or 6 people, accessed 1 to 2 ha of land for cropping, and held between 1 and 5 tropical livestock units (TLU, where 1 is equal to one cow of 250 kg or five goats) (Table 1). In both panels, more prosperous households tended to have less household members, more land, and more livestock. Comparing between the panels, there is evidence of change in the variables associated with prosperity. According to the ANOVA and Tukey's posthoc tests, in the earlier panel, the higher stratum held significantly more land and livestock than the others. In the latter panel this was no longer the case: no differences were found between the high and medium strata at the 5% level of probability for land and livestock (Table 1).

**Table 1:** Farm description variables (medians) for each prosperity strata in the first and second panel. The letters show results of pairwise comparison of ANOVA between strata within the same panel survey (i.e. no comparison has been made between the first and second panel surveys), using Tukey HSD method, where different letters signify differences at the 5% level of probability (*p* ≤0.05).

Panel	Prosperity	Household members	Land owned (ha)	Livestock (TLU)
First	Low	5^a^	0.8^a^	0.9^a^
First	Med	6^a^	1.2^a^	1.1^a^
First	High	5^b^	1.4^b^	3.6^b^
Second	Low	6^a^	1.1^a^	1.4^a^
Second	Med	6^a^	1.6^a,b^	5.1^b^
Second	High	5^b^	1.6^b^	5.0^b^

#### 3.1.3 Food production

The higher value production systems of the more prosperous households are evident when considering the total value of farm produce in the entire study population (measured in local cash value). The higher strata produced vastly more than the other two: in the first panel higher prosperity households produced 66% of all food stuffs, and in the second panel they produced 76% (see Table 2). While higher prosperity households owned more land, they also achieved considerably higher value-production on that land.

**Table 2:** Proportion of land controlled and food production for each of the prosperity strata, in the first and second panel.

Panel	Prosperity	% of population	% of land	% of value of farm produce	Ratio % value farmproduce:% of land
First	Low	30	19	10	0.53
First	Med	33	31	24	0.77
First	High	37	50	66	1.32
Second	Low	27	14	6	0.43
Second	Med	33	29	19	0.66
Second	High	41	57	76	1.33

#### 3.1.4 Changes in livelihoods

It is also necessary to consider the absolute value of farm produce and off-farm incomes, at household level (Table 3). The local market value of all crop and livestock produce, whether eaten or sold, was calculated and any off-farm incomes summed. In the first panel, the three prosperity strata frequently showed significant differences in incomes, but in the second panel the significant differences were more frequent between the high prosperity group and the other two groups. The higher stratum became relatively more productive and more prosperous compared to the medium and low strata. The households in the higher stratum achieved a greater intensity in value crop production ($ value/ha) and off-farm earnings ($ value/person) compared to the others.

**Table 3:** Household performance metrics: median total values of crops produced, total value of livestock products produced, and total value of off farm incomes. Efficiency measures are also provided as an indication of intensification - i.e. producing more with from the same amount of basic resource (land for crops, livestock for livestock products, and people for paid work). The letters show results of pairwise comparison of ANOVA between prosperity strata within the same panel survey (i.e. no comparison has been made between the first and second panel surveys), using Tukey HSD method, where different letters signify differences at the 5% level of probability (*p* ≤0.05). One Tropical Livestock Unit (TLU) means the metabolic equivalent of one cow of 250 kg; Male Adult Equivalent persons (MAE), is a measure of the calorie demand by household members.

Panel	Stratum	Crop Value ($/yr)	Crop Intensity ($/ha/yr)	Livestock Value ($/yr)	Livestock Intensity ($/TLU/yr)	Off Farm Income ($/yr)	Off Farm Income Intensity ($/MAE/yr)
First	Low	325^a^	430^a^	45^a^	70^a^	75^a^	19^a^
First	Med	936^b^	681^b^	131^b^	138^a^	301^b^	73^a^
First	High	1545^c^	931^c^	697^c^	202^a^	1031^c^	343^b^
Second	Low	287^a^	334^a^	0^a^	0^a^	0^a^	0^a^
Second	Med	566^a^	334^a^	288^b^	62^a,b^	420^a^	93^a^
Second	High	1581^b^	872^b^	507^c^	103^b^	1953^b^	522^b^

**Fig. 2:**
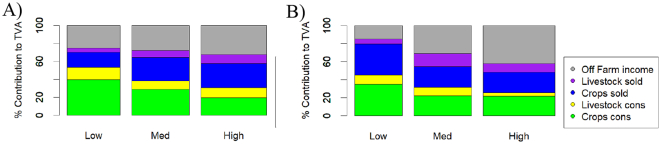
Average proportion of livelihoods derived from sales and consumption of crops, sales and consumption of livestock products, and off-farm incomes. Total Value of Activities (TVA) means the value of all farm produce sold, plus the value of all farm produce consumed, plus the value of all off-farm incomes. A) First Panel; B) Second Panel.

The average proportion of the total household value of activities derived from crops consumed, crops sold, livestock produce consumed, livestock produce sold, and off-farm income is presented in Fig. 2, per prosperity strata. In the earlier panel, the main trends were the increasing role of crop sales, livestock sales, and off-farm income with increasing prosperity. In the latter panel these trends had changed. Off-farm income accounted for a much greater share of the livelihoods of more prosperous households than previously, livestock sales accounted for a greater proportion of livelihood value in the medium strata, and the low prosperity households relied more heavily on crop sales than before.

### 3.2 Household dynamics

#### 3.2.1 Movement between prosperity strata

**Fig. 3:**
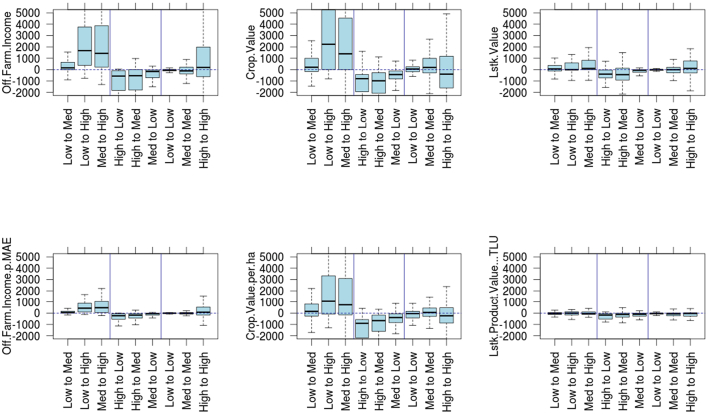
Net change in value of activities (upper panel) and intensity of activities (lower panel), between the first and second panel survey. The upper row shows values at the household level per year, and the lower row shows changes in efficiency of production: off farm incomes per person (measured as male adult equivalent person), crop productivity per hectare of land cultivated, and livestock income per head of livestock owned (normalised using Tropical Livestock Units) All values reported are US$ PPP per household per year. The vertical lines indicate the position of “rising”, “falling” or “steady” households. The horizontal dashed line indicates the location of ‘0’, or no net change.

The “trajectory” of each household was defined by their prosperity stratum in the first panel and again in the second panel (e.g. Low to Low, Low to High) (i.e., see Fig. 1). The nine trajectory groups were approximately equally sized, at around 50 households each, except for the High to Medium and High to High groups, which were around 100 households each (see Table S3 for precise numbers). Fig. 3 and Table S4 present the change in total household value of activities derived from crops, livestock and off-farm for each trajectory group (upper panel), and the intensity of value derived per hectare, per TLU, or per person (lower panel). Rising households (Low to Medium, Low to High, Medium to High) increased the total value from crops and from off-farm sources, and increased the intensity of value-crop production per hectare and off-farm earning per person. Households who moved up to the highest stratum showed very large increases in both absolute value and intensity of crop value production, and off-farm activities, whereas households that moved into the medium class showed more modest gains. The gains from cropping activities were generally larger than those from off-farm activities, but both were of similar magnitude. Although increases in livestock produce value were also evident, they were smaller and no increase in intensity was observed. Falling households showed the inverse: decreased value of crop production and off-farm incomes, accompanied by a reduction in intensity. Livestock product value also dropped for those falling from the high stratum, possibly demonstrating wholesale of livestock as a coping strategy.

Households who remained in the same prosperity strata between the two surveys showed less change in values of production and off-farm incomes than rising or falling households. The households in the Medium-to-Medium trajectory showed a modest increase in crop-value production, whereas the households in the High to High trajectory showed a substantial increase in off-farm incomes and modest decreases in crop production values, perhaps indicating increased focus on off-farm incomes.

#### 3.2.2 Characterizing prosperity strata trajectory groups

Households from the different prosperity strata trajectory groups varied significantly in terms of household welfare indicators from the second panel (unfortunately data for these indicators were unavailable for the first panel) (Fig. S2 and Table S5). The High-to-High trajectory group tended to attain the best welfare indicator scores among all groups having the fewest amount of lean months, experiencing least food insecurity, having a more diverse diet, being least likely to be characterized as suffering from poverty under the PPI index, and generating the largest gross income. This contrasted with the findings for the Low-to-Low trajectory, where households in this group tended to score the worst for these welfare indicators.

Similarly, the High-to-High trajectory group had the highest proportion of households that had attained secondary education or post-secondary education (36%), while it had the fewest households that were illiterate (8%) (Table S6). On the other hand, the Low-to-Low trajectory group had the joint highest proportion of households that were illiterate (24%) and was among the trajectory groups with the fewest households attaining secondary education or higher (16%).

Comparing the trajectory groups in terms of productive assets based on data from the first panel, households from the Low-to-Low trajectory group reported the least amount of land owned (a median of 0.7 ha) and the least livestock owned (median of 0.7 TLUs). The High-to-High trajectory group in comparison owned the most land (1.8 ha) and the second most livestock (3.4 TLUs) (Table S7). In terms of off-farm income, the same pattern was apparent where the Low-to-Low trajectory group generated the least (USD$16 per year), while the High-to-High group generated the second highest amount of off-farm income (USD$444 per year).

#### 3.2.3 Associations between household characteristics and productive assets, and income types

In order to test whether certain household characteristics or base levels of productive assets were associated with higher levels of farm and off-farm in the second panel, linear regression analyses were conducted. Education level attainment displayed a clear trend with both farm and off-income whereby, generally, the higher the level of education attainment the greater the income from either farm or off-farm income (Table S8). This finding suggests that education may play an important role in enabling rural households to generate greater farm income or access improved opportunities for enhanced off-farm income.

The linear regressions applied to assess the relationship between farm income and the baseline levels of productive assets (first panel) revealed significant positive correlations between land and livestock ownership for both the first and second panels. The relationship between farm income and off-farm income was not found to be significant at the 5% level of probability (Table S9). These results indicate that baseline levels of productive assets are not only related to baseline levels of farm income, but also levels of farm income in the second panel.

## 4 Discussion

### 4.1 Pathways out of poverty: Enhanced farm value production

We observed that it was possible for some households to substantially raise their prosperity through enhanced farm value production (Fig. 3). Within the timeframe of this study, it was not clear if this represented a transitory improvement or a more permanent state. While it was not possible to directly assess whether this increase in farm value production was a result of changes in market prices, crop or livestock substitutions to higher value products, or enhanced yields (intensification), similar market prices observed in each panel (Tables S10a-f) and similar frequencies of the main crops grown by those households that rose in prosperity strata (Table S11) suggest that agricultural intensification could have played an important role. The increase in farm value production to lift households above the poverty line was similar to those values calculated from secondary data (Harris, 2019)⁠: a median increase in crop value per hectare of around $1000 (or $2000 per household, see Table S4); and we observed similar losses for households who fell below the poverty line.

Importantly, we observed that households who remained in the lowest prosperity stratum (Low to Low trajectory group) displayed the lowest levels of assets (land owned, livestock owned, and off-farm income) among all trajectory groups (Table S7). This may suggest that a minimum level of productive resources is needed for a farming household to be able to raise their prosperity via enhancing their farm value production. This interpretation is further supported by the finding that farm income from the second panel displayed a positive correlation with the baseline levels of productive assets (land and livestock owned) from the first panel (Table S9).

Low resource endowment as a barrier to poverty alleviation through enhanced farm production has been observed in other studies (Gebreselassie, 2006; van der Lee et al., 2018; Waithaka et al., 2006). For example, in a study in northern Ghana, low resource-endowed farming households were found to exhibit fewer opportunities for saving and investment, restricting their livelihood objectives to subsistence farming and the maintenance of current prosperity levels (Kuivanen et al., 2016). While farming households with fewer resources may be averse to investing in improved agricultural techniques, it seems that their better-off neighbours may be more open to experimenting with innovations, a strategy that may help them generate greater income and thereby lay the pathway to greater prosperity (Jambo et al., 2019; Kurgat et al., 2018).

### 4.2 Pathways out of poverty: Off-farm income activities

The role off-farm income plays in alleviating rural poverty has been debated and theorised in cross-sectional studies (Ellis, 2008; Haggblade et al., 2010; Pender et al., 2006)⁠ but not quantified in a panel survey such as this⁠. Our results provide further evidence that off-farm income in rural areas in developing contexts has the potential to contribute to poverty alleviation by increasing household incomes and enhancing livelihood diversification opportunities (Caulfield et al., 2021; de Sherbinin et al., 2008; Radel et al., 2019).

Off-farm income has been found to alleviate poverty through two main mechanisms, either through re-investment in agricultural intensification (Baltenweck et al., 2003; Bhandari and Ghimire, 2016; Caulfield et al., 2019; Gray and Bilsborrow, 2014) or by simply enhancing the financial resource base of rural households (Jokisch, 2002; Qin, 2010). While our results found evidence that enhanced off-farm income generation helped lift households out of poverty (Fig. 2, Fig. 3, Fig. S4), we found no evidence of a direct relationship between off-farm income and farm income (Table S9). This suggests that households in our sample that generated important amounts of off-farm income may not have been be stimulated to re-invest the additional financial resources from their off-farm activities into farm production, although further research is recommended to explore this relationship in greater detail.

Instead, it may be that these households are “stepping-out” of their farming activities (Dorward et al., 2009). In these cases, such households may be generating sufficient high value off-farm income that lowers dependency on farm income leading to a de-intensification of farming activities (Benayas et al., 2007; Qin, 2010). This is further supported in our findings in that the High-to-High trajectory group experienced an overall decrease in crop value production over the period between surveys (Fig. 3). It is important to point out however, that in many cases this de-intensification of farm activities does not necessarily lead to permanent rural out-migrations. Instead, such households often perceive their off-farm activities as mechanisms to remain in their rural communities (Mata-Codesal, 2018; Ye, 2018).

We also observed that the value of off-farm income was significantly less in households in the low prosperity stratum (especially those in the Low-to-Low trajectory group – Table S7) compared to households in the medium and high strata (Table 3). This mirrored the findings for crop and livestock value production, which were generally much lower in the low prosperity strata (and the Low-to-Low trajectory group) compared to the higher prosperity strata (Table 3).

As argued in others studies, these findings seem to suggest that the most vulnerable farming households are often compelled to explore low-value off-farm livelihood activities in order to compensate for and diversify from their low farm value production levels (Alary et al., 2022; Caulfield et al., 2021). It is likely that these factors are leading to a poverty trap where low value farm production means that households in the low prosperity category must engage in low value off-farm income activities to supplement their livelihoods, subsequently decreasing labour availability and constraining the farming household's capacity to intensify their agricultural production (Barrett and Carter, 2013; Tittonell and Giller, 2013).

This potential poverty trap appears to be further compounded by the finding that the Low-to-Low trajectory group scored among the worst in the household welfare indicators and were among the least educated (Tables S5 and S6 respectively). The latter finding in particular appears to pose a structural barrier to the poorest households from “off-farm” pathways out of poverty as our results indicated that higher levels of off-farm income were mainly associated with higher levels of education attainment (Table S8). Indeed, this relationship between off-farm income activities and education has been found in other studies (Adeoye et al., 2019; Anang and Yeboah, 2019). For example, in a study in Vietnam, education was found to have a significant association with the ability of rural households to generate off-farm income, which in turn was associated with improved welfare indicators and less poverty (Duong et al., 2021).

### 4.3 Policy implications

The persistently poor households in this study were not able to enhance farm value production or generate high value off-farm income, and showed signs of the low productivity-low income poverty trap (Barrett et al., 2001) ⁠, whereby due to very low farm productivity households are forced to sell their labour at low prices in order to survive. More appropriate interventions for such households might be injections of cash, or the development of more sophisticated social safety nets, rather than agricultural support (Banerjee et al., 2019; Gitter et al., 2017). Further research to explore the factors that are associated with households dropping down into deeper poverty levels is therefore highly recommended in order to better develop these social safety nets programs.

Conversely, wealthier households achieved greater gains through greater farm value production and off-farm income. This could be due to their improved access to productive assets, capital and education, and subsequently their greater latitude to innovate and take risks. Regardless of the mechanisms, we observed that enhanced farm value production was mainly practised by the more prosperous households (some of whom had become prosperous through enhanced farm value production). Enhanced farm value production, either through intensification or substitution of farm products with higher yielding or value products, only appears to be a viable strategy to lift already more resource endowed households out of poverty. As discussed by Waithaka et al. (2006) therefore, we suggest that efforts to alleviate poverty through enhancing farm value production should be directed to these wealthier, “medium prosperity” smallholders as they are more likely to have the capacity to intensify.

Furthermore, our results revealed the important role that off-farm activities play in shaping the welfare of rural households, as well as the opportunities and challenges they face. Additional financial resources from off-farm income activities have been found to enable farmers to further intensify agricultural activities or “step-out” of farming while remaining in their rural communities. These trends have important consequences in terms of natural resource management. While de-intensification may present a certain set of opportunities and challenges, (unsustainable) agricultural intensification has the potential to lead to significant land and soil degradation processes (Caulfield et al., 2019; Jaquet et al., 2016). These important trends and processes must therefore be better integrated into rural development policies and programmes in order to better manage land-use transitions in the future.

## 5 Conclusion

We assessed the poverty dynamics of 600 rural households in East Africa across two instances in time. The analyses revealed that enhanced farm value production and off-farm income generation as pathways out of poverty were only open to already more resource endowed households (in terms of access to financial, farm, land, and education resources). On the other hand, less resource endowed households were constrained in their ability to alleviate poverty and were likely caught in a poverty trap. We argue that these results indicate that rural development policies and projects that aim to stimulate farm value production as a means to alleviate poverty will only be successful if directed toward “medium prosperity” rural households, as they have the capacity to enhance farm value production. Conversely, the alleviation of extreme poverty should focus on different means such as cash injections, or the development of more sophisticated social safety nets. Furthermore, we argue that the increasing influence of off-farm income on farm management in rural areas in developing contexts present important opportunities and challenges in terms of natural resource management. These must be better integrated into rural development policies and projects in the future in order to better manage land-use transitions.

## Research ethics statement

This study conforms with the principles of the 1964 WMA declaration of Helsinki. Approval for survey data collection was obtained from project leadership at the International Livestock Research Institute, who provided guidelines for study procedures. Survey participants were not particularly vulnerable, data was processed in anonymised form, and survey participants had the possibility to skip questions. Explicit oral informed consent was obtained from all survey participants prior to survey enumeration and documented as opening question in both surveys. If consent was denied, enumeration stopped after that one question. Permission for obtaining oral rather than written consent from survey respondents was granted, given literacy limitations among the target population.

## Declaration of Competing Interest

None.

## Data Availability

Raw data for all surveys is available, with personally identifiable information removed. The IMPACT-Lite surveys are published with supporting information online at the ILRI Data Portal (http://data.ilri.org/portal/dataset?q=impact+lite). The datasets do not have associated DOIs. The raw data and supporting information for the RHoMIS surveys available in anonymized form (https://doi.org/10.7910/DVN/TFXQJN), and described in van Wijk et al. 2020. Computer code (written in the R language) to process the survey data and repeat the analyses conducted in this manuscript is available from the authors upon request.

## Appendix A Supplementary data

Supplementary material Image 1
