# Supplementary material for: Poverty dynamics and the determining factors among East African smallholder farmers
Source: Agric Syst. 2023 Mar;206:103611. doi: 10.1016/j.agsy.2023.103611 (PMC10009503; doi:10.1016/j.agsy.2023.103611)
Supplement: Supplementary file 1 — Supplementary material [file mmc1.docx]

**Supplementary Information for Poverty Dynamics and the Determining Factors amongst East African Smallholder Farmers**

**Figure S1.** Household welfare indicators for the three prosperity classes, from the second panel survey. The letters show results of pairwise comparison of anova between prosperity clusters within the same panel survey, using Tukey HSD method, *p*<0.05. The welfare indicators were calculated from independent data to that used to generate the prosperity groups, therefore they can be used to verify the prosperity groupings. From left to right and top to bottom, the indicators are: the Probability of Poverty Indicator (higher score means more likely to be poor); the number of months when acquiring sufficient food is a problem; the Household Food Insecurity of Access Scale (higher number means more experiences of hunger); the Households Dietary Diversity Score during the months when food is more available (where a higher number indicates a more diverse diet); and the Households Dietary Diversity Score during the months when food is scarce.

**
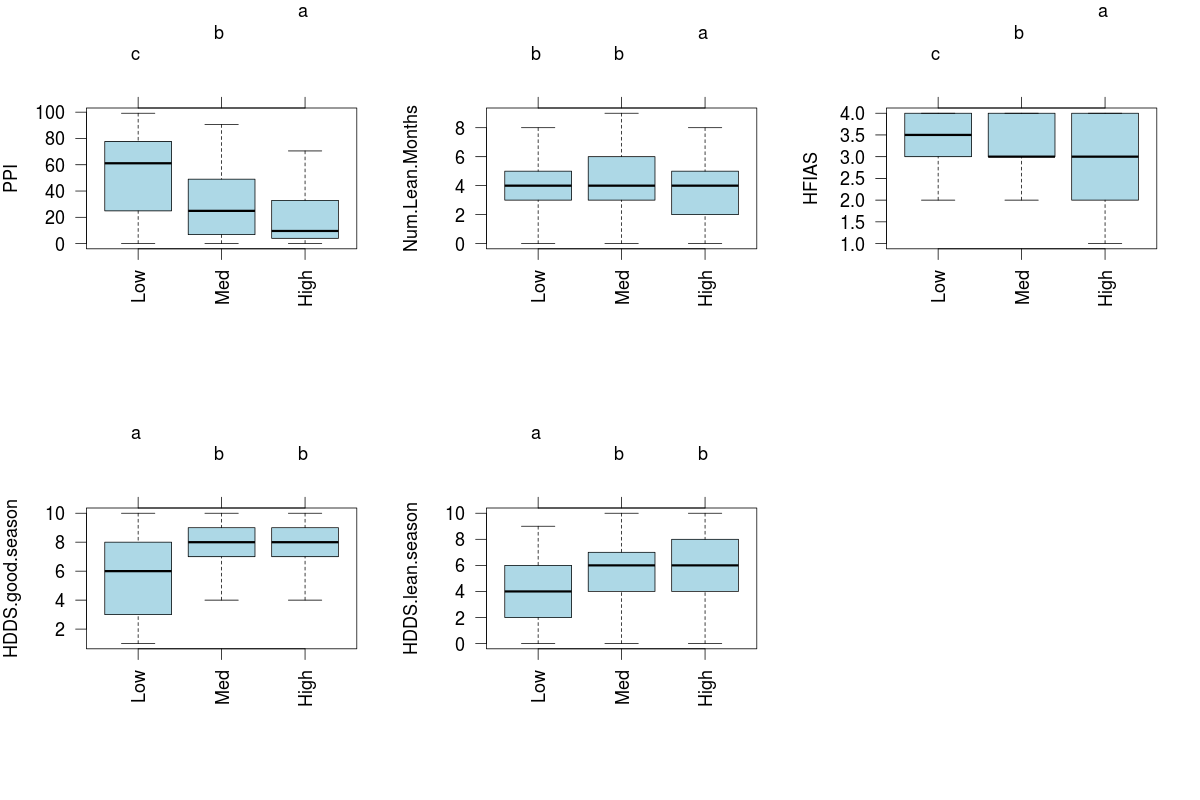
**

**Figure S2.** Household welfare indicators (in the latter panel) for each of the trajectory groups identified. The indicators are the same as described in Fig S1, with the addition of gross household income, which is measured in USD per person per day.


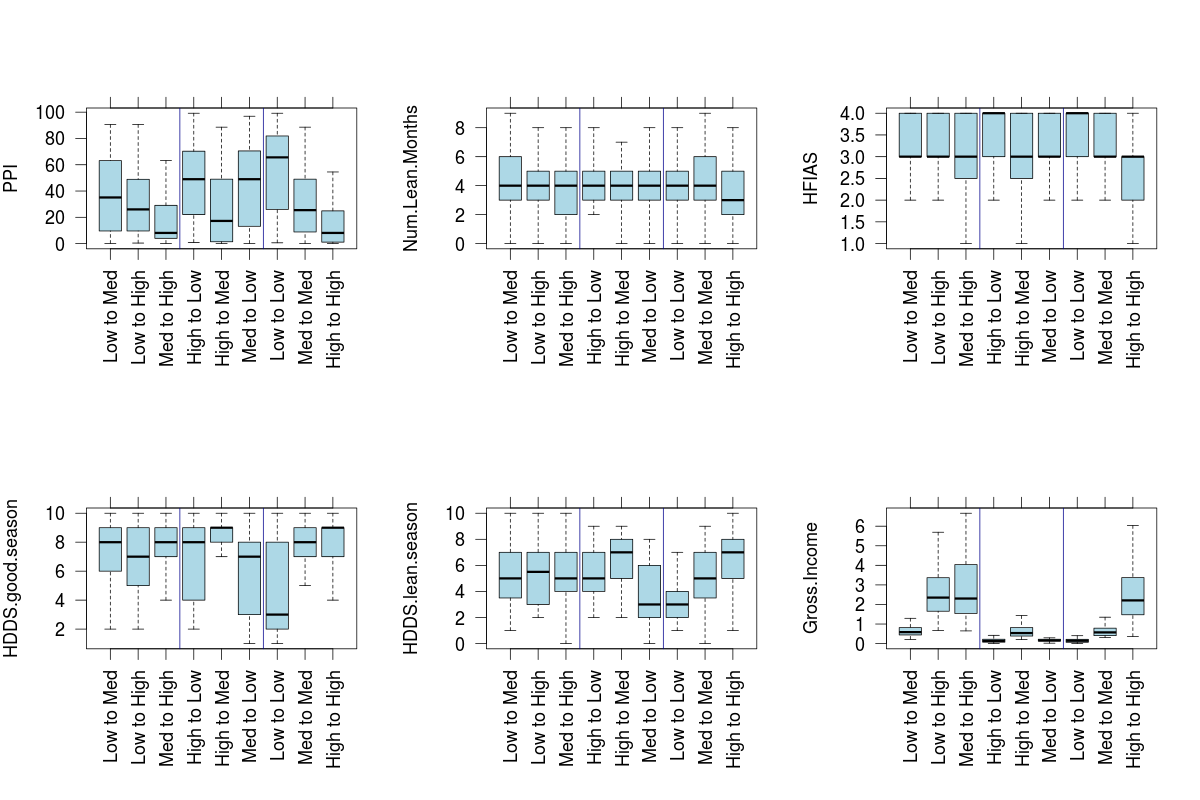


**Figure S3.** Histograms of net change in different farming household resources from the first and second panel surveys by trajectory group. A) household size, B) land owned, C) livestock owned, and D) off-farm income.

A)


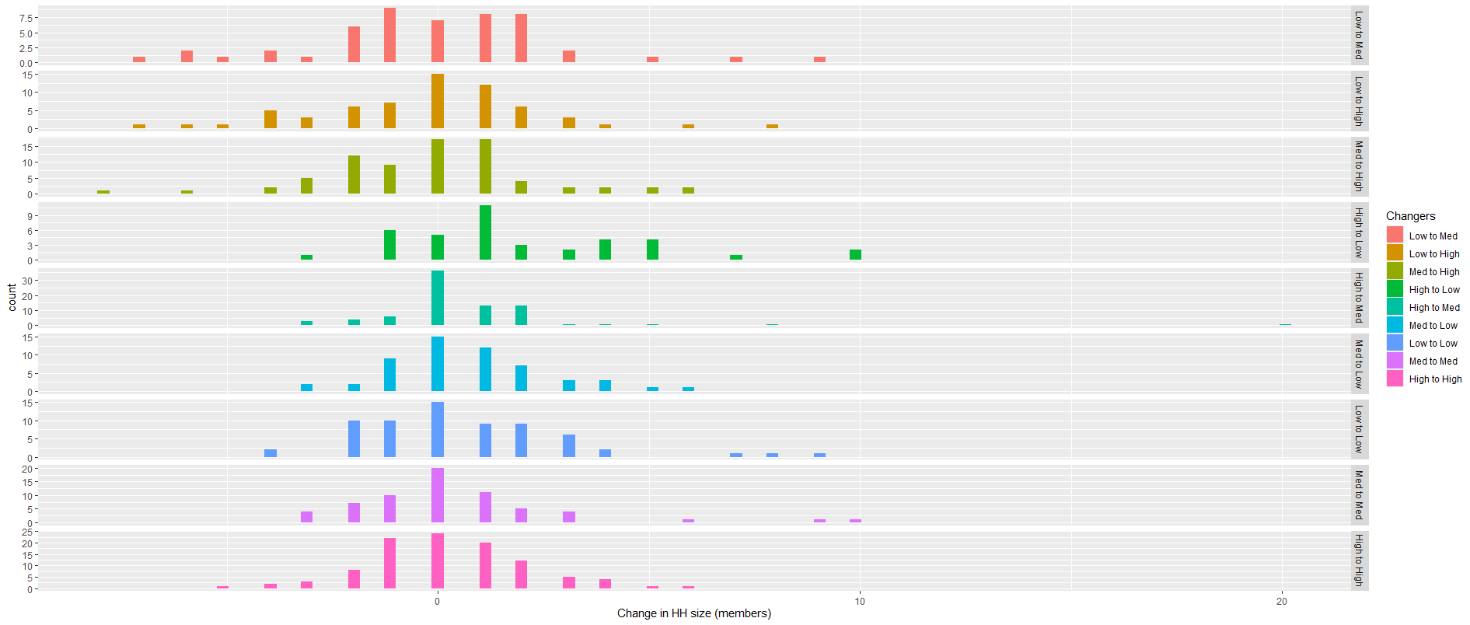


B)


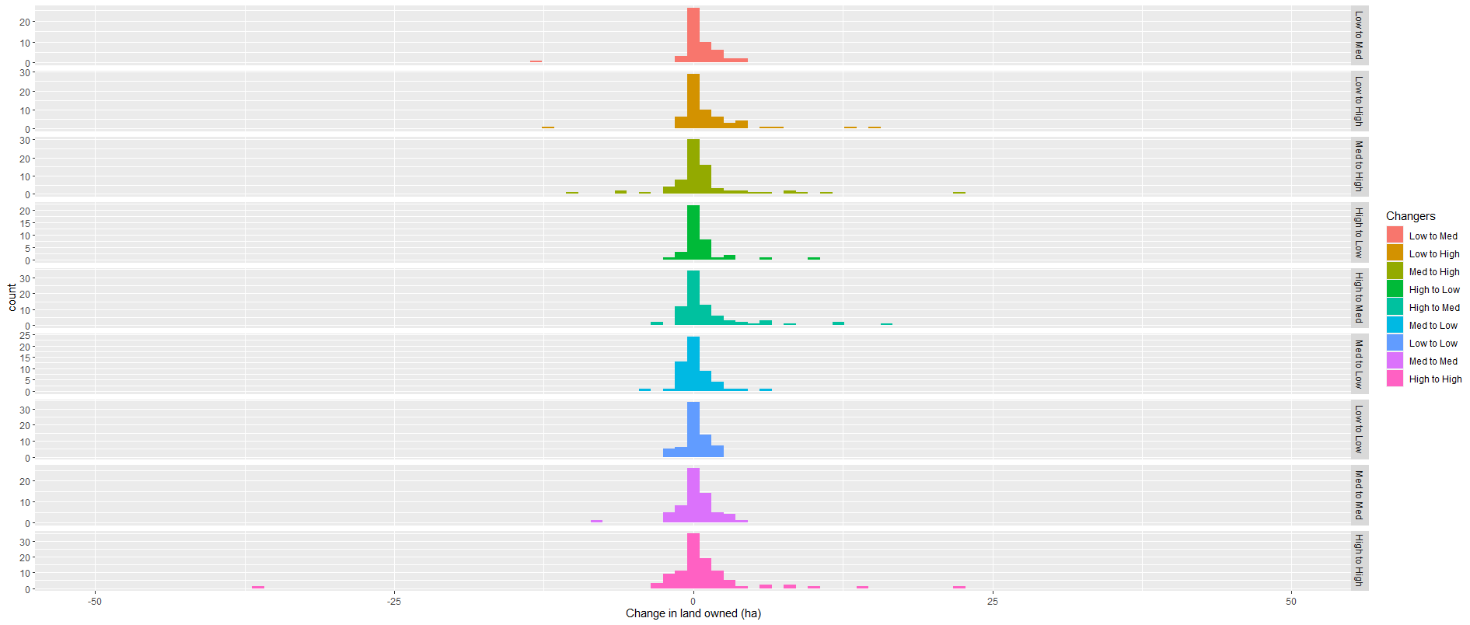


C)


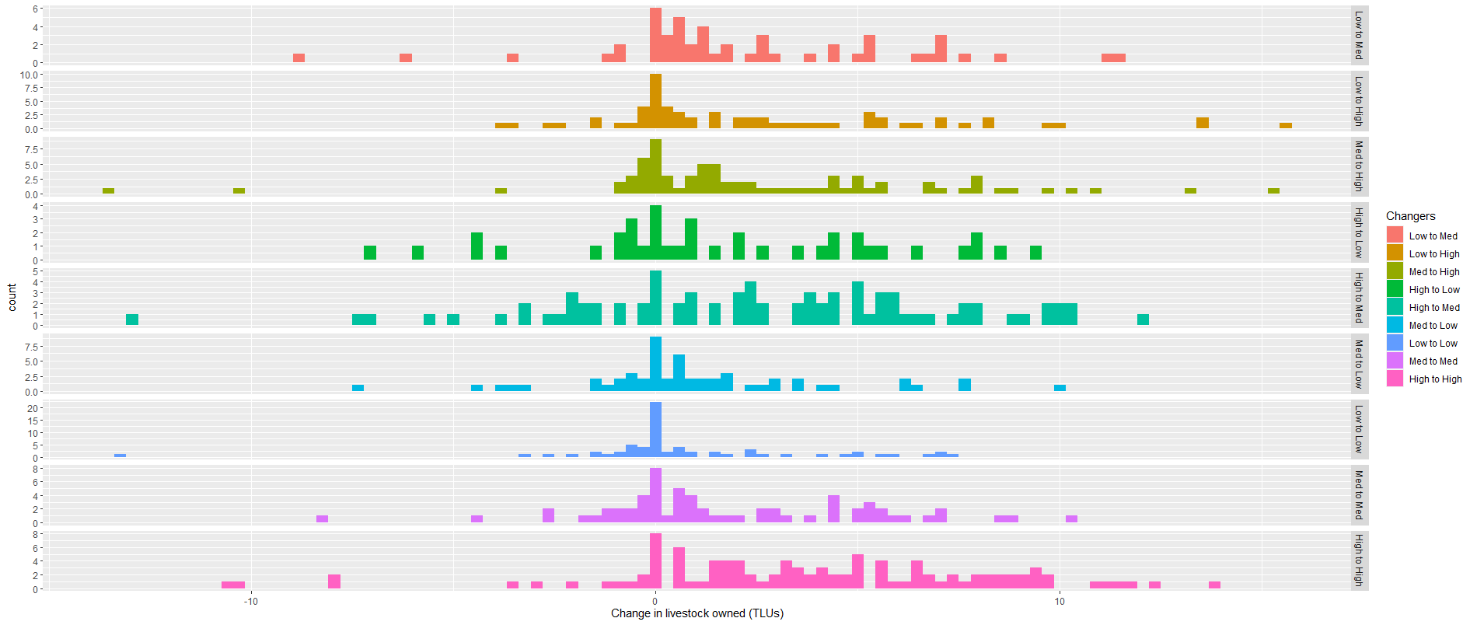


D)


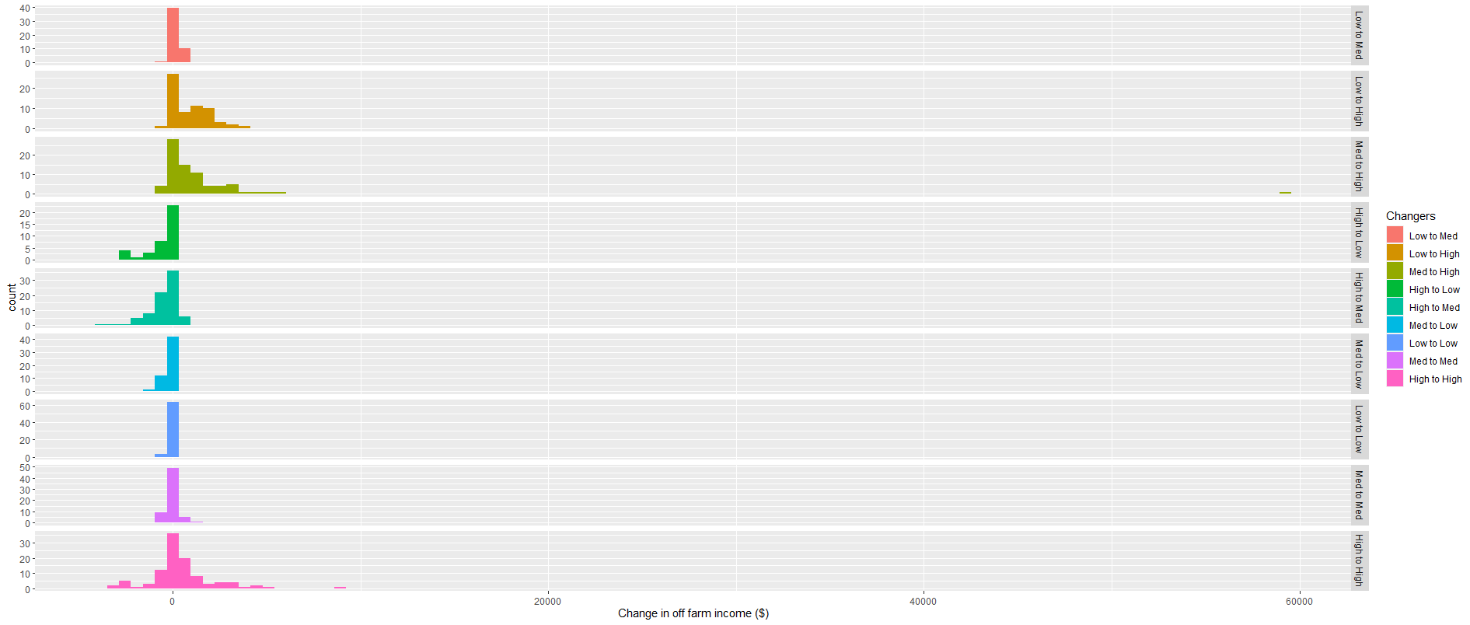


**Figure S4.** Proportion of households growing the most common crops (10% of households or more) of households that have moved up in prosperity strata (“Risers” – Low-to-Medium, Low-to-High, Medium-to-High). A) 1^st^ panel, B) 2^nd^ panel.


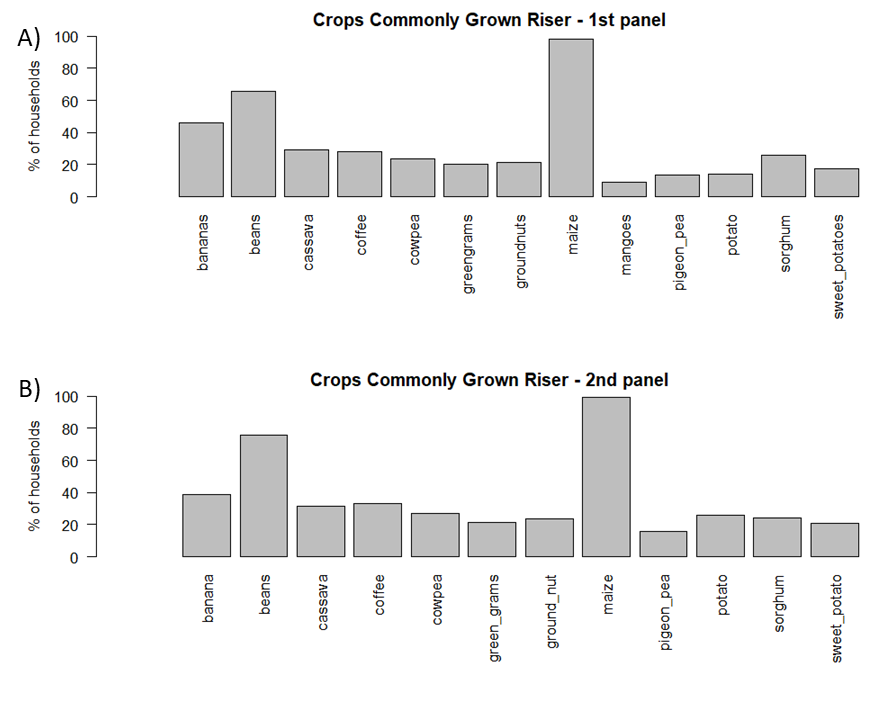


**Table S1a**: Sale prices (USD$) of main crops sold by households in the Kenyan datasets for panel 1 and panel 2

| Crop | Both Kenyan sites - 1st panel | Nyando - 2nd panel | Wote - 2nd panel |
| --- | --- | --- | --- |
| banana | 0.17 | 0.25 | - |
| beans | 0.65 | 0.59 | 0.59 |
| cassava | 0.41 | 0.10 | - |
| cowpea | 0.44 | 0.22 | 0.22 |
| green grams | 0.59 | 0.70 | 0.70 |
| ground nut | 1.18 | 0.50 | - |
| maize | 0.46 | 0.25 | 0.25 |
| mango | 0.08 | 0.08 | 0.08 |
| millet | 0.63 | 0.19 | 0.19 |
| oranges | 0.06 | 0.16 | 0.16 |
| pigeon_pea | 0.47 | 0.60 | 0.60 |
| sorghum | 0.46 | 0.20 | 0.20 |
| tomato | 0.40 | 0.50 | 0.50 |
| onion | 0.59 | 0.25 | - |

**Table S1b**: Sale prices (USD$) of main livestock products sold by households in the Kenyan datasets for panel 1 and panel 2

| Livestock | Product | Nyando | | Wote | |
| --- | --- | --- | --- | --- | --- |
|  |  | 1st panel | 2nd panel | 1st panel | 2nd panel |
| cattle | milk | 0.53 | 0.25 | 0.47 | 0.25 |
| chicken | eggs | 0.12 | 0.10 | 0.12 | 0.10 |
| goats | milk |  | 0.25 |  | 0.25 |
| cattle | wholesale |  | 200 |  | 250 |
| chicken | wholesale | 3.53 | 5 | 3.53 | 5 |
| goats | wholesale |  | 90 |  | 30 |
| sheep | wholesale |  | 175 |  | 50 |
| donkeys horses | wholesale |  | 2800 |  | 100 |

**Table S1c**: Sale prices (USD$) of main crops sold by households in the Tanzanian datasets for panel 1 and panel 2

| Crop | 1st panel | 2nd panel |
| --- | --- | --- |
| banana | 0.19 | 0.05 |
| beans | 0.76 | 0.65 |
| cabbage | 0.13 | 0.19 |
| coffee | 1.27 | 0.83 |
| fruits | - | 0.26 |
| maize | 0.35 | 0.32 |
| plantain | - | 1.61 |
| potato | 0.21 | 0.25 |
| sugarcane | 0.06 | 0.19 |
| tomato | 0.16 | 0.33 |
| vegetables | 0.18 | 0.26 |

**Table S1d**: Sale prices (USD$) of main livestock products sold by households in the Tanzanian datasets for panel 1 and panel 2

| Livestock | Product | 1st panel | 2nd panel |
| --- | --- | --- | --- |
| cattle | milk | 0.25 | 0.40 |
| chicken | eggs | 0.13 | 0.05 |
| chicken | wholesale | 1.27 |  |

**Table S1e**: Sale prices (USD$) of main crops sold by households in the Ugandan datasets for panel 1 and panel 2

| Crop | 1st panel | 2nd panel |
| --- | --- | --- |
| banana | 0.07 | 0.40 |
| beans | 0.29 | 0.59 |
| cabbage | 0.24 | 0.50 |
| cassava | 0.05 | 0.20 |
| coffee | 0.30 | 0.80 |
| cowpea | 0.05 | 0.22 |
| egg plants | 0.01 | 0.50 |
| ground nut | 0.06 | 0.50 |
| maize | 0.20 | 0.25 |
| mango | 0.20 | 0.08 |
| millet | 0.21 | 0.19 |
| onions | 0.18 | 0.40 |
| potato | 0.05 | 0.20 |
| sorghum | 0.05 | 0.20 |
| sugar cane | 0.32 | 0.20 |
| sweet potato | 0.06 | 0.20 |
| tobacco | 0.40 | 1.00 |
| tomato | 1.08 | 0.50 |

**Table S1f**: Sale prices (USD$) of main livestock products sold by households in the Ugandan datasets for panel 1 and panel 2

| Livestock | Product | 1st panel | 2nd panel |
| --- | --- | --- | --- |
| cattle | milk | 0.20 | 0.25 |
| chicken | eggs | 0.12 | 0.10 |
| goats | milk |  | 0.25 |
| cattle | wholesale |  | 250 |
| chicken | wholesale | 1.21 | 5 |
| goats | wholesale |  | 30 |
| sheep | wholesale |  | 50 |
| donkeys | wholesale |  | 100 |

**Table S2.** Median and standard deviation (in parentheses) of household welfare indicators for the three prosperity classes, from the second panel survey (see Figure S1). The indicators presented include: the Probability of Poverty Indicator (higher score means more likely to be poor); the number of months when acquiring sufficient food is a problem; the Household Food Insecurity of Access Scale (higher number means more experiences of hunger); the Households Dietary Diversity Score during the months when food is more available (where a higher number indicates a more diverse diet); and the Households Dietary Diversity Score during the months when food is scarce.

| Prosperity strata | Num. lean months | HFIAS | HDDS flush season | HDDS lean season | PPI |
| --- | --- | --- | --- | --- | --- |
| Low | 4 (1.8) | 4 (0.7) | 6 (2.9) | 4 (2.2) | 61 (31.0) |
| Med | 4 (2.4) | 3 (0.8) | 8 (1.9) | 6 (2.1) | 25 (26.9) |
| High | 4 (2.4) | 3 (1.0) | 8 (2.2) | 6 (2.2) | 10 (23.5) |

**Table S3.** The number of respondents in each of the “trajectory” groupings (i.e. relating to changed poverty strata between the first and second panel survey rounds).

| Trajectory | Number of observations |
| --- | --- |
| Low to Low | 66 |
| Low to Med | 50 |
| Low to High | 63 |
| Med to Low | 39 |
| Med to Med | 43 |
| Med to High | 57 |
| High to Low | 55 |
| High to Med | 101 |
| High to High | 122 |

**Table S4.** Change in median farm performance variables between the first and second panel, per trajectory grouping.

| Trajectory | Crop Value ($/yr) | Crop Intensity ($/ha/yr) | Lstk Value ($/yr) | Lstk Intensity ($/TLU/yr) | Off Farm Income ($/yr) | Off Farm Income Intensity ($/MAE/yr) |
| --- | --- | --- | --- | --- | --- | --- |
| Low to Med | 207 | 147 | 57 | -43 | 156 | 47 |
| Low to High | 2229 | 1048 | 2 | -13 | 1673 | 437 |
| Med to High | 1393 | 736 | 105 | -43 | 1430 | 474 |
| High to Low | -793 | -903 | -395 | -175 | -567 | -233 |
| High to Med | -982 | -666 | -442 | -124 | -533 | -181 |
| Med to Low | -433 | -371 | -54 | -104 | -161 | -48 |
| Low to Low | 50 | -43 | -6 | -28 | -26 | -9 |
| Med to Med | 189 | 53 | -4 | -67 | -110 | -22 |
| High to High | -397 | -229 | 108 | -40 | 192 | 74 |

**Table S5.** Median Household welfare indicators (in the latter panel – first panel data unavailable) followed by standard deviation (in parentheses) for each of the trajectory groups identified. The letters show results of pairwise comparison of anova between prosperity clusters within the same panel survey, using Tukey HSD method, different letter means differences at the 5% level of probability. The indicators are the same as described in Figure S2, with the addition of gross household income, which is measured in USD per person per day. The indicators presented include: the Probability of Poverty Indicator (higher score means more likely to be poor); the number of months when acquiring sufficient food is a problem; the Household Food Insecurity of Access Scale (higher number means more experiences of hunger); the Households Dietary Diversity Score during the months when food is more available (where a higher number indicates a more diverse diet); and the Households Dietary Diversity Score during the months when food is scarce.

| Trajectory | Num. lean months | HFIAS | HDDS flush season | HDDS lean season | PPI | Gross income |
| --- | --- | --- | --- | --- | --- | --- |
| Low to Med | 4 (2.1)ab | 3 (0.7)bc | 8 (2.5)bcd | 5 (2.2)bc | 35 (30)cd | 0.6 (0.3)a |
| Low to High | 4 (1.9)ab | 3 (1.0)bc | 7 (2.5)bc | 6 (2.0)c | 26 (26)bcd | 2.4 (1.9)b |
| Med to High | 4 (2.6)ab | 3 (0.9)b | 8 (1.9)cd | 5 (2.2)cd | 8 (22)ab | 2.3 (5.5)b |
| High to Low | 4 (1.6)ab | 4 (0.7)bc | 8 (2.7)bc | 5 (2.2)cd | 49 (31)de | 0.1 (0.1)a |
| High to Med | 4 (2.1)ab | 3 (0.9)b | 9 (1.4)d | 7 (1.7)d | 17 (27)ac | 0.5 (0.1)a |
| Med to Low | 4 (2.0)ab | 3 (0.8)bc | 7 (2.9)ab | 3 (2.3)ab | 49 (32)de | 0.2 (0.3)a |
| Low to Low | 4 (1.9)ab | 4 (0.6)c | 3 (2.8)a | 3 (1.6)a | 66 (30)e | 0.1 (0.1)a |
| Med to Med | 4 (3.0)b | 3 (0.8)bc | 8 (2.0)cd | 5 (2.1)c | 25 (24)bc | 0.6 (0.3)a |
| High to High | 3 (2.4)a | 3 (1.0)a | 9 (2.1)cd | 7 (2.2)d | 8 (21)a | 2.2 (2.2)b |

**Table S6.** Proportion (%) of households reaching different education attainment levels by trajectory group

| Trajectory | Illiterate | literate | primary | secondary | post-secondary |
| --- | --- | --- | --- | --- | --- |
| Low to Med | 22 | 2 | 70 | 6 | 0 |
| Low to High | 24 | 8 | 56 | 13 | 0 |
| Med to High | 12 | 9 | 57 | 20 | 3 |
| High to Low | 21 | 3 | 62 | 10 | 5 |
| High to Med | 18 | 1 | 52 | 24 | 5 |
| Med to Low | 11 | 4 | 74 | 9 | 2 |
| Low to Low | 24 | 2 | 59 | 14 | 2 |
| Med to Med | 11 | 6 | 55 | 23 | 5 |
| High to High | 8 | 2 | 55 | 24 | 12 |

**Table S7.** Median farming household assets from the first panel by trajectory grouping. Standard errors are presented in parentheses next to the mean. Letters to the right of the standard error indicate results of a Fisher’s Least Significance Difference Test, with different letters indicating at the 5% level of probability (p-value = 0.05)

| Trajectory | Household Members | Land Owned (ha) | Livestock (TLU) | Off-farm income ($/yr) |
| --- | --- | --- | --- | --- |
| Low to Med | 7 (2.0)^a^ | 0.8 (2.5)^de^ | 1.5 (2.8)^cd^ | 51 (119)^cde^ |
| Low to High | 6 (2.2)^ab^ | 0.9 (1.7)^de^ | 1.0 (2.7)^cd^ | 31 (116)^de^ |
| Med to High | 6 (2.4)^ab^ | 1.5 (2.1)^abc^ | 1.1 (3.4)^bcd^ | 99 (283)^cd^ |
| High to Low | 4 (2.6)^ab^ | 1.1 (0.8)^bcde^ | 2.8 (3.1)^abc^ | 286 (801)^abc^ |
| High to Med | 5 (2.2)^ab^ | 1.4 (1.5)^ab^ | 4.2 (4.1)^a^ | 621 (770)^a^ |
| Med to Low | 6 (2.0)^ab^ | 1.0 (0.9)^cde^ | 1.3 (2.3)^cd^ | 86 (296)^bcd^ |
| Low to Low | 5 (2.0)^ab^ | 0.7 (0.8)^e^ | 0.7 (2.3)^d^ | 16 (131)^e^ |
| Med to Med | 6 (2.2)^a^ | 1.2 (2.1)^bcd^ | 0.8 (2.5)^cd^ | 177 (288)^cde^ |
| High to High | 5 (2.1)^b^ | 1.8 (5.8)^a^ | 3.4 (3.6)^ab^ | 444 (953)^ab^ |

**Table S8.** Annual farm and off-farm income ($USD year^-1^) by education level attainment and panel. Standard errors are presented in parentheses next to the mean. Letters to the right of the standard error indicate results of a Fisher’s Least Significance Difference Test, with different letters indicating at the 5% level of probability (p-value = 0.05).

| Farm or off-farm income | Panel | illiterate | literate | primary | secondary | post-secondary |
| --- | --- | --- | --- | --- | --- | --- |
| Off-farm  income | First | 52 (16)^ab^ | 9 (5)^a^ | 54 (9)^b^ | 74 (22)^bc^ | 424 (250)^c^ |
|  | Second | 16 (7)^a^ | 18 (15)^ab^ | 32 (7)^ab^ | 48 (18)^ab^ | 263 (203)^b^ |
| Farm  income | First | 43 (12)^a^ | 64 (35)^ab^ | 79 (11)^a^ | 253 (68)^b^ | 89 (48)^ab^ |
|  | Second | 142 (21)^a^ | 256 (75)^abc^ | 226 (17)^b^ | 359 (51)^c^ | 396 (114)^bc^ |

**Table S9.** Correlations between productive assets and farm income (USD$ year^-1^). Productive assets were assessed based on the data from the first panel to assess whether these baseline assets were correlated with higher farm income in the first and second panels

| Productive asset | Panel | Coefficient | p-value |
| --- | --- | --- | --- |
| Livestock ownership (TLUs) | First | 0.19 | <0.001 |
|  | Second | 0.06 | 0.002 |
| Land owned (ha) | First | 0.03 | <0.001 |
|  | Second | 0.04 | 0.022 |
| Off-farm income (USD$ year^-1^) | First | <0.001 | 0.074 |
|  | Second | <0.001 | 0.206 |
